# Prediction of quality of life in schizophrenia using machine learning models on data from *Clinical Antipsychotic Trials of Intervention Effectiveness* (CATIE) schizophrenia trial

**DOI:** 10.1038/s41537-022-00236-w

**Published:** 2022-03-21

**Authors:** Mélissa Beaudoin, Alexandre Hudon, Charles-Edouard Giguère, Stéphane Potvin, Alexandre Dumais

**Affiliations:** 1 Centre de Recherche de l’Institut Universitaire en Santé Mentale de Montréal, Montreal, Canada; 2 Department of Psychiatry and Addictology, Faculty of Medicine, Université de Montréal, Montreal, Canada; 3 Faculty of Medicine, McGill University, Montreal, Canada; 4 Institut National de Psychiatrie Légale Philippe-Pinel, Montreal, Canada

**Keywords:** Schizophrenia, Human behaviour

## Abstract

While research focus remains mainly on psychotic symptoms, it is questionable whether we are placing enough emphasis on improving the quality of life (QoL) of schizophrenia patients. To date, the predictive power of QoL remained limited. Therefore, this study aimed to accurately predict the QoL within schizophrenia using supervised learning methods. The authors report findings from participants of a large randomized, double-blind clinical trial for schizophrenia treatment. Potential predictors of QoL included all available and non-redundant variables from the dataset. By optimizing parameters, three linear LASSO regressions were calculated (*N* = 697, 692, and 786), including 44, 47, and 41 variables, with adjusted R-squares ranging from 0.31 to 0.36. Best predictors included social and emotion-related symptoms, neurocognition (processing speed), education, female gender, treatment attitudes, and mental, emotional, and physical health. These results demonstrate that machine learning is an excellent predictive tool to process clinical data. It appears that the patient’s perception of their treatment has an important impact on patients’ QoL and that interventions should consider this aspect.

**Trial registration:** ClinicalTrials.gov Identifier: NCT00014001.

## Introduction

Schizophrenia is a chronic and severe mental disorder that can be invalidating^1^. This disorder can greatly affect the quality of life (QoL)^2,3^, which is defined by the World Health Organization as an individuals’ perception of their position in life in the context of the culture and value systems in which they live and in relation to their goals, expectations, standards, and concerns^4^.

A shift has recently been observed in the objectives of schizophrenia treatment. While the goal was once to reduce symptoms only, this has changed to focus more on recovery through improving QoL and functioning^5,6^. Although complete recovery is often not possible for these patients, they can still recover in some way. This notably involves optimizing their well-being and functioning, which are key components of QoL. Over the past few years, factors that may promote better QoL have been identified in the literature, with mixed results. Some predictors that recur frequently are types of psychiatric or psychotic symptoms, but which type exactly predicts best QoL remains controversial^2,7–10^. These can be reduced by using medication; however, even though response and adherence to antipsychotics can improve QoL^3^, some medication side effects such as weight gain^11^ and sexual dysfunction^12^ have been associated with a worsened outcome. Other predictors of higher QoL were also identified, e.g., a better cognition and an older age of disorder onset^13–15^. On the other hand, stigma-related feelings and comorbid diagnoses predicted a poorer outcome regarding QoL^14^. In general, it seems that the highly heterogeneous factors presented in the current literature largely depend on the angle from which the authors choose to approach the question. Another issue is that the design is often cross-sectional, which does not allow for longitudinal predictions. Identifying the most important and essential factors could help identify which patients are better able to recover, and ultimately optimize every patient’s recovery.

Several researchers have used multivariate models to predict the quality of life. Mohamed et al.^13^ created a model excluding variables that may be redundant with QoL (e.g., functioning) using longitudinal data from the Clinical Antipsychotic Trials of Intervention Effectiveness (CATIE) schizophrenia study. In doing so, they were able to explain 22% of the variance in total QoL with positive and negative symptoms, neurocognitive, and sociodemographic variables (age, race, ethnicity, gender, and time). In such studies, the explained variance is generally low^2,14^, possibly because authors did not include some factors that deviate from their research question and that may play a major role in QoL (e.g., physical health, patients’ self-reported satisfaction, and medication adherence). With the emergence of supervised machine learning, it now becomes possible to reach an optimal model including the best predictors among fairly large datasets, and without human a priori in the way variables are combined^16,17^. This new approach could thereby provide a better understanding of the various factors that influence QoL in individuals with schizophrenia, just as it successfully predicted other outcomes such as relapses^18,19^.

The aim of the current study was to identify, using machine learning, factors that predict QoL among people with schizophrenia. To do so, we computed important variables from the CATIE study, a large naturalistic clinical trial conducted between 2000 and 2004 in the United States.

## Results

### Sample characteristics

Due to attrition and missing data, only 919 of the 952 participants with a longitudinal follow-up were included in a model (*N* = 697, 692, and 786 in models 1–3, respectively). From this number, 670 were males (73%) and the average age was of 41.1 years (SD = 11.0; range: 18–67). One-quarter did not complete high school (25%), a minority was employed full-time at the time of the study (6%), and only a few were married (11%). Most of the sample had no comorbid psychiatric condition (60%). Detailed baseline sample characteristics were presented in Table 1. At the baseline, 6-month and 12-month follow-up visits, the QoL total score was on average 2.8 (SD = 1.1), 2.9 (SD = 1.1, and 3.0 (SD = 1.1), respectively.

**Table 1:** Baseline sample characteristics. *N* = 919.

Baseline dichotomous characteristics	N/mean	%/SD	Minimum	Maximum
Male gender	670	72.9	–	–
Married	103	11.2	–	–
Veterans	**1**97	21.4	–	–
Living with a significant other	163	17.7	–	–
Did not complete high school	225	24.5	–	–
Employed full-time	58	6.3	–	–
*Ethnicity*				
White	576	62.7	–	–
Black	307	33.4	–	–
American Indian or Alaska Native	14	1.5	–	–
Asian	26	2.8	–	–
Hispanic Latino or Spanish Origin	101	11.0	–	–
Hawaiian or Pacific Islander	6	0.7	–	–
*Comorbid psychiatric diagnoses*				
Obsessive–compulsive disorder	40	4.4	–	–
Other anxiety disorder	78	8.5	–	–
Major depression	124	13.5	–	–
Alcohol dependence	77	8.4	–	–
Alcohol abuse	78	8.5	–	–
Drug dependence	63	6.9	–	–
Drug abuse	101	11.0	–	–
Antisocial personality disorder	5	0.5	–	–
Other personality disorder	9	1.0	–	–
Other comorbid diagnosis	37	4.0	–	–
No comorbid condition	552	60.1		
Age	41.1	11.0	18	67
Years of education	11.6	3.4	1	21
Years since first psychiatric treatment	16.7	11.6	0	56
Years since first prescribed antipsychotic medication	14.4	11.1	0	56
*Number of previous hospitalizations*				
Lifetime	2.7	1.5	0	4
Past year	0.6	0.9	0	4
QoL total score	2.8	1.1	0.4	5.9

### Linear regressions using machine learning

Three longitudinal models were calculated to predict QoL (1) 12 months after the baseline, (2) 6 months after the 6-month visit, and (3) 6 months after the baseline.

The first model attempting to predict the 12-month QoL with baseline variables attained an uncentered adjusted *R*-squared of 0.350 and comprised 45 predictors. All included variables and associated coefficients are presented in Table 2. The mean squared error (MSE) training result was 0.92 and the MSE testing result was 0.97.

**Table 2:** Linear regression of QoL at the 12-month visit using baseline variables. *N* = 697.

Categories	Baseline variables	Coeff.
Sociodemographics	• Parents highest education level	0.3359
	• Veteran	−0.1633
	• Male gender	−0.1597
	• Hispanic, Latino, or Spanish origin	0.1159
	• Race: white	−0.0065
Psychiatric diagnoses	• Major depression	-0.1057
	• No comorbid psychiatric conditions	0.0287
	• Other diagnoses	−0.0238
	• Alcohol abuse	−0.0201
Positive and negative symptoms scale (PANSS)	Negative symptoms:	
	• Emotional withdrawal	−0.6451
	• Passive apathetic social withdrawal	−0.5087
	General symptoms:	
	• Poor attention	−0.0739
Calgary depression rating scale (CDRS)	• Hopelessness	−0.2367
	• Self-depreciation	0.1284
Neurocognitive battery	• Processing speed score	0.6454
	• Working memory score	0.1433
	• Verbal score	0.0068
Clinical Global Impressions Scale (CGIS)	• Patient-reported mental/emotional health	0.4272
	• Number of days smoking cigarettes in the past week	−0.2416
	• Clinician global impression of severity	−0.2062
	• Productive activities are [x] time more important than least important CGIS item	0.2010
	• Medication side effects	0.1585
	• Energy and interests	0.1541
	• Disturbing and unusual experiences	−0.1336
	• CGIS Response	0.1305
	• Alcohol consumption	0.1031
	• Energy and interests are [x] time more important than least important CGIS item	0.0996
	• Medication side effects are [x] time more important than least important CGIS item	0.0386
	• Satisfaction of contact with mental health professionals	0.0321
Insight and Treatment Attitudes Questionnaire (ITAQ)	• Do you have mental problems?	0.2525
	• Will you take the medication?	0.1867
	• Have you had mental problems that were different from most other people’s?	0.0613
Drug Attitude Inventory (DAI)	• Staying on meds prevent me from getting sick	0.1515
	• I feel weird like a zombie on meds	−0.0333
	• Meds make me feel tired and sluggish	0.0176
Physician’s assessment of the severity of the adverse event	• Sleepiness	0.1005
Patient’s assessment of the severity of the adverse event	• Sleepiness	0.1689
	• Sexual arousal	0.0606
	• Weight gain	0.0163
Antipsychotic medication	• No antipsychotic medication	−0.0679
	• Risperidone	0.0270
	• Other antipsychotics	−0.0140
Laboratory values	• Mean corpuscular hemoglobin	0.0936
Other variables	• Medical history status	0.0478
	• Day screened (vs baseline)	−0.0022

As for the second model predicting the 12-month QoL using variables from the 6-month visit, the optimal regression (Table 3) comprised 47 predictors, and the uncentered adjusted *R*-squared was 0.365. The MSE training result was 0.86 and the MSE testing result was 0.98.

**Table 3:** Linear regression of QoL at the 12-month visit using variables from the 6-month visit. *N* = 692.

Categories	6-months variables	Coeff.
Sociodemographics	• Parents highest education level	0.2722
	• Veteran	−0.2634
	• Patient’s highest education level	0.2596
	• Male sex	−0.0704
	• Race: black of African American	0.0076
Positive and negative symptoms scale (PANSS)	Positive symptoms:	
	• Grandiosity	0.1114
	• Hallucinatory behavior	−0.0431
	Negative symptoms:	
	• Passive apathetic social withdrawal	−0.7232
	• Emotional withdrawal	−0.5901
	• Poor rapport	−0.3056
	General symptoms:	
	• Active social avoidance	−0.4386
	• Guilt feelings	0.1710
	• Anxiety	0.1508
Calgary depression rating scale (CDRS)	• Hopelessness	−0.3738
Neurocognitive battery	• Processing speed standardized to baseline	0.2795
	• Neurocognitive composite score standardized to baseline	0.2301
	• Vigilance score standardized to baseline	0.1303
	• Verbal score standardized to baseline	0.0844
Clinical global impressions scale (CGIS)	• Clinician global impression of severity	−0.4592
	• Satisfaction of contact with mental health professionals	0.2716
	• Patient version, clinical global impression of severity	−0.2379
	• Patient-reported mental/emotional health	0.2259
	• Energy and interests	0.1678
	• Productive activities	−0.1248
	• Tobacco products use	−0.1020
	• Energy and interests are [x] time more important than least important CGIS item	0.0884
	• CGIS response	0.0799
	• Disturbing and unusual experiences are [x] time more important than least important CGIS item	0.0392
	• Alcohol use	0.0340
Insight and treatment attitudes questionnaire (ITAQ)	• Do you now need to take medication for mental problems?	0.2900
Drug attitude inventory (DAI)	• I feel weird like a zombie on meds	0.0880
	• Medication is unnatural for my mind and body	−0.0473
	• The good of meds outweighs the bad	0.0006
Physician’s assessment of the severity of the adverse event	• Sialorrhea	0.1961
	• Hypersomnia	−0.0667
	• Akinesia	0.0101
Impact of adverse event on patients’ adherence to medication	• Akinesia	0.3743
	• Dry mouth	0.2459
	• Weight gain	0.2242
	• Sialorrhea	0.0255
Antipsychotic medication	• Adherence^a^	0.0630
	• Total # of days taking olanzapine (between baseline and the 6-month visit)^a^	−0.0616
	• Has the patient taken quetiapine (between baseline and the 6-month visit)^a^	−0.0427
	• Total # of days taking risperidone (between baseline and the 6-month visit)^a^	0.0051
Laboratory values	• Total bilirubin level	0.5947
	• HDL cholesterol level	0.0862
Other variables	• Childhood antisocial behaviors	−0.0427

^a^Variables that have only been measured during follow-up visits (not during the baseline visit), and that therefore could only be a predictor in this model.

Finally, the QoL at 6 months was estimated using baseline variables in a third model. With 41 variables, an uncentered adjusted *R*-squared of 0.307 was obtained. The complete model and its parameters are presented in Table 4. The MSE training result was 0.93 and the MSE testing result was 0.96.

**Table 4:** Linear regression of QoL at the 6-month visit using baseline variables. *N* = 786.

Categories	Baseline variables	Coeff.
Sociodemographics	• Male gender	−0.3105
	• Parents highest education level	0.2604
	• Patient’s highest education level	0.2491
	• Veteran	−0.1065
	• Hispanic, Latino, or Spanish origin	0.0380
Psychiatric diagnoses	• No comorbid psychiatric conditions	0.2136
Positive and negative symptoms scale (PANSS)	Positive symptoms;	
	• Hostility	−0.0038
	Negative symptoms;	
	• Passive apathetic social withdrawal	−0.7467
	• Stereotyped thinking	−0.2305
	• Poor rapport	−0.1324
	• Emotional withdrawal	−0.0737
	General symptoms;	
	• Lack of judgment and insight	−0.0751
	• Somatic concern	−0.0139
	• Active social avoidance	−0.0131
Calgary depression rating scale (CDRS)	• Hopelessness	−0.0562
Neurocognitive battery	• Processing speed score	0.3155
	• Working memory score	0.1009
	• Neurocognitive composite score	0.0934
Clinical global impressions scale	• CGIS response	0.2470
	• Productive activities are [x] time more important than least important CGIS item	0.2123
	• Patient-reported mental/emotional health	0.1501
	• Energy and interests	0.0477
	• Alcohol use	0.0266
	• Disturbing and unusual experiences	−0.0219
Insight and treatment attitudes questionnaire (ITAQ)	• Do you at any time need treatment, hospitalization, or outpatient care?	0.1624
	• Do you now need to take medication for mental problems?	0.1114
	• Have you at any time needed to take medication for mental problems?	0.0689
	• How much information did you recently receive from mental health service providers?	0.0398
Drug attitude inventory (DAI)	• Staying on meds prevent me from getting sick	0.0470
	• My thoughts are clearer on medication	0.0401
	• Good outweighs the bad	0.0282
	• Medication is unnatural for my mind and body	−0.0117
	• I feel more normal on medication	−0.0104
	• Meds make me feel tired and sluggish	0.0089
Physician’s assessment of the severity of the adverse event	• Sexual orgasm	−0.0766
Patient’s assessment of the severity of the adverse event	• Weight gain	0.1433
	• Insomnia	−0.0514
Antipsychotic medication	• Olanzapine	0.0606
	• No antipsychotic medication	−0.0466
Other variables	• Medication switch status	0.0160
	• Day screened (vs. baseline)	0.0032

A summary of the results of the three prediction models is presented in Table 5. Among the strongest and most reliable predictors were having low/no passive apathetic social withdrawal, low/no emotional withdrawal, and having a high processing speed score. Many other variables were also present in all three models, including having educated parents, self-reporting high mental health, female gender, being treatment-responsive (CGIS), gaining weight as a side-effect, and having energy and interests. Being a veteran and being hopeless were negatively associated with QoL. Other predictors were strong but only present in one or two models; having a high level of total bilirubin, a higher education level, or believing that they had a mental problem was associated with a better QoL. Meanwhile, having a high clinical global impression of severity, social avoidance, poor rapport, stereotyped thinking, and dry mouth as a side-effect was associated with poorer outcomes.

**Table 5:** Summary of variables favoring quality of life. Variables with a similar meaning (e.g., different scales for the same side effect) were merged. Predictors are presented in order of effect sizes.

Predictors present in all models	Predictors present in two models	Predictors present in only one model
**Low/no passive apathetic social withdrawal**	**Low clinical global impression of severity**	**High total bilirubin**
**Low/no emotional withdrawal**	Having a higher education level	Believing that they have mental/nerve/worry problems
**Neuro: high processing speed score**	Low/no active social avoidance	Having dry mouth as an adverse event
Having more educated parents	Low/no poor rapport	Low/no stereotyped thinking
High patient-reported mental/emotional health	Subjective need to take medication for mental problems	Having akinesia as an adverse event
Not being hopeless	Low/no tobacco use	Saying that they will take the medication
Female gender	A high neurocognitive composite score	Having guilt feelings
Not being a veteran	Being satisfied with providers	Believing that, at any time, they needed treatment hospitalization or outpatient care
CGIS response	Neuro: high working memory score	Having anxiety
More severe weight gain	Having no comorbid condition^a^	Having sleepiness as an adverse event
High/important energy and interests	*Believing that staying on meds prevent them from getting sick*	Neuro: high vigilance score
*High/important productive activities*	*Being of Hispanic, Latino, or Spanish origin*	Low/no self-depreciation
*Consuming alcohol*	*Taking antipsychotics at baseline*	Having grandiosity
*Not having disturbing and unusual experiences or considering them as important*	*Neuro: high verbal score*	Having sialorrhea as an adverse event
	*Not thinking that medication is unnatural for their mind and body*	Not having a diagnosis of major depression^a^
	*Not feeling weird like a ‘zombie’ on medication*	*Having medication side effects*
	*Using risperidone as an antipsychotic*	*High mean corpuscular hemoglobin*
	*Thinking that good things about medication outweigh the bad*	*High HDL cholesterol*
	*Thinking that meds make them feel tired and sluggish*	*Low/no sexual orgasm-related adverse event*
	*Having a longer period between the screening and the baseline visit*	*Not lacking judgment and insight*
		*Low/no poor attention*
		*Believing that, at any time, they needed to take medications for mental problems*
		*Low/no hypersomnia as an adverse event*
		*Good adherence to study medication*^b^
		*Believing that, at any time, they had mental problems that were different from most other people’s*
		*Suffering from sexual arousal-related adverse event*
		*Low/no insomnia as an adverse event*
		*Past/inactive medical Hx status*
		*Low/no hallucinatory behavior*
		*Low/no childhood antisocial behaviors*
		*Not using quetiapine*
		*Thinking that their thoughts are clearer on medication*
		*Having received a lot of information from mental health service providers about the illness*
		*Olanzapine use*
		*Not having another psychiatric diagnosis (apart from abuse/dependence, OCD, anxiety, major depression, and personality disorders)*^a^
		*No alcohol abuse diagnosis*
		*Medication switch status*^a^
		*Not taking another antipsychotic (apart from olanzapine, quetiapine, risperidone, ziprasidone, haloperidol, decanoate, and perphenazine)*^a^
		*Low/no somatic concern*
		*Feeling more normal on medication*
		*Being black or African American*
		*Not being white*
		*Low/no hostility*

^a^Variables that have only been measured during the screening or baseline visit, and that therefore could only be a predictor in models 1 and 3.

^b^Variable that has only been measured during follow-up visits, and that therefore could only be a predictor in model 2.

Bold: coefficient over 0.3.

Italic: coefficient under 0.1.

## Discussion

This study aimed to accurately predict further QoL by identifying the characteristics that make individuals more prone to recover. By using machine learning to create optimal models, good predictions have been reached, and this despite adjustments to avoid any redundancy or collinearity of the data. Three models were calculated: (1) prediction of 12-month QoL with baseline variables, (2) prediction of 12-month QoL with 6-month variables, and (3) prediction of 6-month QoL with baseline variables. *R* squares of 0.350, 0.365, and 0.307 were achieved for each of these models, respectively. Identified predictors included, among others, social and emotion-related symptoms, neurocognition (processing speed), education, female gender, veteran status, indicators of satisfaction with psychiatric treatment as well as elements of physical functioning. The performance of the model is consistent with the prediction score for human behavior modeling^20^.

Firstly, predictors of QoL include many symptoms related to social and emotional aspects of life (e.g., negative association with social and emotional withdrawal, social avoidance, poor rapports, and hopelessness), thereby highlighting the fact that socialization and social roles are central determinants of QoL. Notably, the patients’ and their parents’ education level, likely associated with social inclusion and socioeconomic status, were strong predictors, as previously demonstrated^21^. Similar results have previously been obtained with emotional discomfort^22^. It is indeed possible that the relationship between negative symptoms and the QoL observed in the literature is due to the patients’ ability to interact with others as well as their environment. These factors might be related to social support as well, which is a key component of QoL^23^. The lack of social support is indeed a major problem for individuals with schizophrenia^23^, and it is, therefore, a crucial determinant to consider. Female gender was also associated with higher QoL; this predictor is, however, controversial in the current literature^24–28^. The backgrounds and origins of patients also seem to have an impact, since parental education level and veteran status were among identified predictors. This finding could be linked to the fact that schizophrenia patients with a greater trauma history tend to have a poorer QoL^29^.

Secondly, as previously demonstrated with that database^13^, neurocognition had a significant impact on QoL. Considering each subscale separately, the processing speed was found to be the most predictive, even more than the total neurocognition score. This finding suggests that cognitive rehabilitation programs, which have already proven to be effective to improve cognitive performance, symptoms, and psychosocial functioning^30^, could be an important element to improving QoL as well^31^.

Many subjective factors were also classified as very strong predictors of QoL. For example, good mental health, evaluated by the physician or reported by the patient, was contributing to a favorable outcome. Satisfaction toward mental health providers was also an important predictor, which was previously shown to be associated with a better QoL^32^. This finding suggests that the patients’ subjective satisfaction is a very important factor when it comes to recovery. Additionally, having a good attitude toward the medication (e.g., thinking that medication is needed or that it prevents them from getting sick) also seemed important. These factors are likely to be associated with better medication adherence, as supported by other recent studies of people with schizophrenia^15,33^. Adherence was only found to be a weak predictor in one model; however, it should be noted that it was only a potential predictor in the second model as this was not measured at the baseline visit, since the patients were not yet taking the study medication. Antipsychotic medication is indeed considered important to improve the mental health of schizophrenia patients. However, while they contribute to the improvement of the symptomatology, they also cause a lot of side effects, thereby having contradictory effects on QoL. In the current study, side effects and treatment attitudes seemed more important than specific drugs, demonstrating that the ideal medication varies from patient to patient, and that adherence and observed changes are more important in predicting QoL. Nevertheless, response to treatment, measured using the CGIS questionnaire, was found to be a strong predictor in all three models. These results confirm those of Naber et al., who came to similar conclusions using the CATIE database^34^.

Finally, some physical health indicators were included in the models (e.g., bilirubin). Physical comorbidities being very frequent in that population could reflect the presence of metabolic disorders that greatly impact the QoL of some individuals. Tobacco use, which is well established to be associated with significant physical disorders, was also a predictor in two models. Similarly, predictors related to adverse events were also probably associated with physical health, which is unsurprisingly a great predictor of QoL in schizophrenia^35^. However, weight gain was found to be predictive of a better QoL in all models. This result is controversial since that side-effect is usually associated with poorer outcomes. However, compliant patients might be at higher risk of gaining weight from medication, which could explain that association^36^.

Although this study innovates by demonstrating that QoL can be predicted effectively in schizophrenia patients, a few limitations must be acknowledged. Despite that the prediction was great in that cohort, it is not necessarily representative of the overall schizophrenia population. Subjects were excluded if they had certain psychiatric comorbid diagnoses that are fairly frequent in that population (e.g., mental retardation and schizoaffective disorders), and they were all willing to participate as well as able to provide informed consent. However, this is an issue that is common to all randomized controlled trials, and the researchers minimized that issue by including a large number of sites representative of the United States population. Nevertheless, more such studies will be needed to confirm the predictors identified. This model could also be tested on another population to assess to what extent it is generalizable.

In conclusion, this study allowed an excellent prediction of the QoL of patients with schizophrenia using machine learning algorithms. Among the best and most reliable predictors of QoL were notably characteristics linked to social and emotional symptoms, good attitude toward medication, satisfaction toward healthcare providers and patients’ own mental health, neurocognition, female gender, and medication side-effects. Since good prediction levels can be achieved, the use of machine learning could have major implications for the future of prediction as it helps avoid human bias. Eventually, it will also become possible to create predictive algorithms that could be used on various clinical populations and guide clinicians in their decision-making. The study of the predictors identified by such algorithms also allows a bit more insight into how a disease such as schizophrenia manifests itself and into the mechanisms that could explain the outcome. Notably, in the present study, we were able to identify very precise symptoms and factors that could have a higher impact than expected on the QoL of people with schizophrenia (e.g., their subjective perception of their mental health). In doing so, it was notably observed that physical health variables, which are often omitted from mental health-related studies, seem to have an important impact on schizophrenia patients’ QoL. Consequently, interventions aiming to increase QoL should also consider these aspects. More studies will be needed to confirm the results and their applicability for clinicians.

## Methods

### Study sample

Data for this study were extracted from the CATIE schizophrenia study dataset. CATIE was a large, naturalistically designed clinical trial conducted by the *National Institute of Mental Health* (NIMH) between December 2000 and December 2004. 1460 patients with a DSM-IV diagnosis of schizophrenia, based upon the Structured Clinical Interview for DSM-IV^37^, were followed for 18 months. The trial was approved by the institutional review board at each site, and the patients or their legal guardians provided their written informed consent. The detailed study description and design can be found elsewhere^38^.

A subsample of 952 patients was selected based on the longitudinal monitoring of their QoL, i.e., they had completed at least 2 visits among the baseline visit and the 6, 12, and 18-month follow-up visits. According to the protocol, participants should have been followed for 18 months, with a follow-up visit occurring every 3 months or so. However, the attrition rate was very high, and therefore some variables were missing for some participants. Consequently, only data up to 12 months were used, and 697 subjects could be included in the first model, whereas the second and the third comprised 692 and 786 individuals, respectively.

### Dataset

The QoL was measured every 6 months using a well-validated clinician-rated scale, the *Heinrichs-Carpenter Quality of Life Scale* (QOL)^39^. The objective was to use total QoL score at 6 and 12 months as a continuous outcome, i.e., the dependent variable, while all other variables from the CATIE trial were used as potential predictors in linear regressions. These included a large number of questionnaire items as well as the total scores and other variables (dichotomous or continuous) that were in the database, for a total of 253 potential baseline predictors and 233 potential 6-month predictors. Notably, psychotic symptoms were accessed during each visit using the positive and negative syndrome scale^40^. Depressive symptoms were measured every 3 months using the Calgary depression rating scale^41^. Neurocognition was measured using a neurocognitive battery accessing verbal learning, vigilance, speed, reasoning, and working memory. Other potential predictors were selected based on what was available within the database. These included many variables, both demographic and clinical, and both psychiatric and somatic (e.g., sociodemographic variables, metabolic biomarkers, complete blood count, side effects severity, antipsychotic medication, insight and attitudes toward treatment, adherence, violence, drug use, general status, vitals, etc.). However, items that were considered too conceptually related to the concept of QoL (i.e., redundant with items of the QoL questionnaire) were removed from the database. Included variables were all detailed in the Supplementary Table. In the models where computed not only the scales’ totals but also every single item included in each tool and questionnaire.

### Statistical analysis

A Lasso supervised regularization algorithm was implemented to identify potential predictors for three models: (1) baseline predictors of 12-month QoL, (2) 6-month predictors of 12-month QoL, and (3) baseline predictors of 6-month QoL. This type of regularization regression was developed to enable feature (predictor) selection and regularize the dataset to optimize prediction accuracy. By conducting multiple analyses in parallel, it is possible to assume that the variables that recur consistently across models are probably stronger predictors since these remain important over time.

The Lasso algorithm, from the Sk learn library (version 1.0.1), was implemented in Python 3.9. The train the regularization algorithm, 70% of the dataset was used whereas 30% is used for testing, which performed well in similar studies with datasets of this size in the literature^42,43^. A pre-processing of the data took place prior to this division. Participants for whom 25% of data were missing were removed from the dataset. The remaining missing data was accounted for by using the mean value of the other participants which is a technique called mean imputation often used in order to stabilize the classification process (selection of predictors). This algorithm is consistent with other studies conducted in the field of psychiatry. Best performing hyperparameters were identified using the GridSearchCV algorithm provided by the Sk learn library. An alpha = 0.01, max_iter = 100,000 and default values for the remaining parameters were selected by the GridSearchCV.

The performance of the algorithm for the three models was analyzed as follows. The MSE for the training set and for the testing set were calculated and compared. An R2 score was calculated for both the training set and testing sets. The testing R2 score is representative of our predictive score where a score of 1 would indicate that the model explains all the variation of the dependent variable around its mean compared to a score of 0 which means that the model does not explain at all the observed variations. Collinearity between the different variables is accounted for in the Lasso algorithm by its regulative nature: it keeps all the features of the model but gradually reduces the coefficient up to 0 of variables that are not of interest in the model to predict the dependent variable.

To account for the validation of the regression algorithm over the three models, tenfold cross-validation was conducted. This validation method, which is repeated ten times, divides the dataset randomly into ten parts and nine of those parts are used for training whereas the remaining one is used for testing.

### Reporting summary

Further information on research design is available in the Nature Research Reporting Summary linked to this article.

## Supplementary information

Reporting Summary

Supplementary Table. Variables included into database and computed using supervised machine learning.

Supplementary Method. Pseudocode

## Data Availability

The Clinical Antipsychotic Trials of Intervention Effectiveness Schizophrenia Trial is a limited access dataset available on request, under certain conditions, from the National Institutes of Mental Health clinical trials.

## Code availability

Algorithms were all derived from the Scikit learn library version 1.0.1, a publicly available resource that includes simple and efficient tools for predictive data analysis. These were implemented using Python 3.9. Pseudocode with all relevant parameters was made available; see Supplementary Method. For intellectual property reasons, the complete code will not be made accessible.
