# Supplementary material for: Prediction of quality of life in schizophrenia using machine learning models on data from Clinical Antipsychotic Trials of Intervention Effectiveness (CATIE) schizophrenia trial
Source: Schizophrenia (Heidelb). 2022 Mar 21;8(1):29. doi: 10.1038/s41537-022-00236-w (PMC8938459; doi:10.1038/s41537-022-00236-w)
Supplement: Supplementary file 2 — Supplementary Table. Variables included into database and computed using supervised machine learning. [file 41537_2022_236_MOESM2_ESM.docx]

**Supplementary Table. Variables included into database and computed using supervised machine learning.**

| Variables | Description |
| --- | --- |
| Baseline Sociodemographics | |
| Gender   - Male - Female | Dichotomic : applies (1) vs does not apply (0) |
| Age | Continuous variable |
| Race   - White - Black - American Indian or Alaska Native - Hispanic | Dichotomic : applies (1) vs does not apply (0) |
| Education   1. Education level 2. If did not finish high school, number of years of education 3. Total number of years of education 4. Parents’ highest education level | Items (a) and (c): ordinal   1. Did not complete high school 2. GED/High school diploma 3. Some college, did not graduate 4. Community college or technical school 5. College graduate 6. College graduate and some Master’s level courses 7. Master’s degree completed 8. Advanced degree courses, not graduated 9. Advanced degree completed   Items (b) and (c): continuous |
| Veteran | Dichotomic : applies (1) vs does not apply (0) |
| Spanish speaking patient | Dichotomic : applies (1) vs does not apply (0) |
| Day screened | Day screened related to the baseline. For example, a value of -5 indicate that a patient was screened 5 days prior to the baseline interview. |
| Psychiatric history   1. Age first treated for emotional or behavioral problem 2. Years since first treated for emotional or behavioral problem 3. Age f at first antipsychotic prescription 4. Years since first prescribed antipsychotic medication 5. Number of previous hospitalizations, lifetime 6. Number of previous hospitalizations, past year 7. Recent hospitalization or crisis-stabilization | Items (a) – (f) : continuous scales  Item (g): dichotomic, applies (1) vs does not apply (0). Past three months. |

**Supplementary Table. Variables included into database and computed using supervised machine learning.** *(continued)*

| Variables | Description |
| --- | --- |
| Baseline Sociodemographics *(continued)* | |
| Childhood antisocial behaviors severity | Represent the number of items coded yes (1) vs. no (0) in that questionnaire. All items were asked for before age 15. The severity ranges between 0 and 6.   - Did you skip school a lot? - Did you run away from home overnight more than once? - Did you ever deliberately destroy someone else’s property? - Did you often start physical fights? - Were you ever arrested or sent to juvenile court? - Were you expelled or suspended from school? |
| Antipsychotic medication before the start of the study ^a^   1. Olanzapine 2. Quetiapine 3. Risperidone 4. Ziprasidone 5. Haloperidol 6. Fluphenazine decanoate 7. Perphenazine 8. Other antipsychotics 9. All other antipsychotics 10. No antipsychotic medication 11. Olanzapine monotherapy 12. Quetiapine monotherapy 13. Risperidone monotherapy 14. Combination Meds 15. All other monotherapy 16. Medication switch status | Items (a) – (o): dichotomic, applies (1) vs does not apply (0)  Item (p) : dichotomic, active (1) vs. inactive (0) |
| Physical health   1. Have a hysterectomy 2. Did the hysterectomy include an oophorectomy? 3. Any active significant medical history 4. Tardive dyskinesia | Dichotomic : applies (1) vs does not apply (0)  Item (d): Meet Schooler Kane criteria for probable tardive dyskinesia |

**Supplementary Table. Variables included into database and computed using supervised machine learning.** *(continued)*

| Variables | Description |
| --- | --- |
| Baseline Sociodemographics *(continued)* | |
| Psychiatric comorbid diagnoses:   - Alcohol dependence - Alcohol abuse - Drug dependence - Drug abuse - Obsessive-compulsive disorder - Other anxiety disorder - Major depression - Antisocial personality disorder - Other personality disorder - Other diagnosis - No comorbid condition | Dichotomic : applies (1) vs does not apply (0). Measured during the screening interview using the *Structured Clinical Interview for DSM-IV* (SCID-IV) [1]. |
| Clinical psychiatric factors | |
| Clinical Global Impression Scale (CGIS) [2]   1. Patient version of CGIS 2. Energy and interest 3. Social relations 4. Disturbing and unusual experiences 5. Confusion and difficulty concentrating 6. Medication side effects 7. Productive activities 8. Patient reported mental / emotional health 9. Illness information received 10. Satisfaction with providers 11. Tobacco products 12. Alcohol 13. Marijuana 14. Cocaine 15. Opiates 16. PCP 17. Amphetamine 18. Other substance 19. Cigarettes/day past week 20. Clinician Drug Use Scale 21. Clinician Alcohol Use Scale 22. Clinician Global Impressions of Severity 23. CGIS response | Items (a) and (v): ordinal   1. Normal, Not ill 2. Minimally ill 3. Midly ill 4. Moderately ill 5. Markedly ill 6. Severely ill 7. Very severely ill   For items (b) to (g), patients were asked to rate each item on a scale from 1 (the least important) to 6 (the most important), in addition to decide how much times more important that item is compared to the least important one.  Item (h) is expressed in %  Items (i) and (j): ordinal scale, for past 3 months   1. None at all 2. A little 3. Some 4. A lot   Items (k) to (r) : Yes (1) or No (0) for the past 3 months  Item (s) is a numeric (scale)  Items (t) and (u): ordinal   1. Abstinent 2. Use without impairment 3. Abuse 4. Dependence 5. Dependence with institutionalization   Item (w) : response to medication, yes (1) vs. no (0) |

**Supplementary Table. Variables included into database and computed using supervised machine learning.** *(continued)*

| Variables | Description |
| --- | --- |
| Clinical psychiatric factors *(continued)* | |
| *Calgary Depression Scale* *for Schizophrenia* [3]   1. Depression 2. Hopelessness 3. Self deprecation 4. Guilty ideas of reference 5. Pathological guilt 6. Morning depression 7. Early Wakening 8. Suicide 9. Observed depression 10. Total score | Items (a) to (i): ordinal   1. Absent 2. Mild 3. Moderate 4. Severe   Item (j) : Scale ranging from 0 to 27. A score of 6 or higher is was shown to be predictive of a depressive episode [4]. The Minimum Clinically Important Difference (MCID) for that scale was suggested to be 1.3 [5]; therefore, significant variation in one item can have a clinically significant impact on the patient’s symptomatology. |
| *Drugs Attitude inventory* [6]   1. Good outweighs the bad 2. I feel weird like a zombie on medication 3. I take my medications of my own free choice 4. Medications make me feel more relaxed 5. Medications make me feel tired and sluggish 6. I take medications only when I am sick 7. I feel more normal on medication 8. It is unnatural for my mind and body 9. My thoughts are clear on medication 10. Staying on medications prevent me from getting sick 11. Total score | Items (a) to (j): dichotomic, True (1) vs. False (0)  Item (k) : scale ranging from -10 to 10. A higher score equals a better attitude. |
| *Insight and Treatment Attitudes Questionnaire* [7]   1. Have you at any time had mental (“nerves”, “worry”) problems that were different from most other people's? 2. Have you at any time needed treatment (hospitalization or outpatient care) for mental problems? 3. Do you now have mental problems? 4. Do you now need treatment for mental problems? 5. Is it possible that in the future you may have mental problems? 6. Will you in the future need continued treatment for mental problems? | Items (a) to (k) : scale from 0 to 2, 0 meaning no insight and 2 meaning good insight.  Item (l) : scale ranging from 0 to 22, a higher score equals a better insight. |

**Supplementary Table. Variables included into database and computed using supervised machine learning.** *(continued)*

| Variables | Description |
| --- | --- |
| Clinical psychiatric factors *(continued)* | |
| *Insight and Treatment Attitudes Questionnaire* [7] *(continued)*   1. Have you at any time needed to take medications for mental problems? 2. Do you now take medications for mental problems? 3. Will you in the future need to take medications for mental problems? 4. Will you take the medications? 5. Do the medications do you any good? 6. Total score |  |
| *Positive and Negative Symptoms Scale* [9]   1. P1 – Delusions 2. P2 – Conceptual organization 3. P3- Hallucinatory behavior 4. P4 – Excitement 5. P5 – Grandiosity 6. P6- Suspiciousness persecution 7. P7 – Hostility 8. N1 – Blunted affect 9. N2 – Emotional Withdrawal 10. N3 – Poor rapport 11. N4 – Passive apathetic social withdrawal 12. N5 – Difficulty in abstract thinking 13. N6 – Lack of spontaneity and flow of conversation 14. N7 – Stereotyped thinking 15. G1 – Somatic concern 16. G2 – Anxiety 17. G3 – Guilt feelings 18. G4 – Tension 19. G5 – Mannerism and posturing 20. G6 – Depression 21. G7 – Motor retardation 22. G8 – Uncooperativeness 23. G9 – Unusual thought content 24. G10 – Disorientation 25. G11 – Poor attention 26. G12 – Lack of judgement and insight 27. G13 – Disturbance of volition 28. G14 – Poor impulse control 29. G15 – Preoccupation | Items (a) – (dd) : ordinal   1. Absent 2. Minimal 3. Mild 4. Moderate 5. Moderately severe 6. Severe 7. Extreme   Items (ee) – (gg) are total of subscales. (ee) is the sum of items (a) – (g) and vary between 7 and 49; (ff) is the sum of items (h) – (n) and vary between 7 and 49; (ff) is the sum of items (o) – (dd) and vary between 16 and 112.  Item (hh) is the total score of the scale, representing the sum of items (a) – (dd) and could vary between 30 and 210. A higher total score represents a more severe symptomatology. |

**Supplementary Table. Variables included into database and computed using supervised machine learning.** *(continued)*

| Variables | Description |
| --- | --- |
| Clinical psychiatric factors *(continued)* | |
| *Positive and Negative Symptoms Scale* [9] *(continued)*   1. G16 – Active social avoidance 2. PANSS positive score 3. PANSS negative score 4. PANSS general score 5. PANSS total score |  |
| Neurocognition standardized to baseline score:   - Verbal learning - Vigilance - Speed - Reasoning - Working memory - Total (mean) | The test comprised in the neurocognitive assessment battery were selected carefully by a Neurocognitive Advisory Group and a Neurocognitive Assessment Unit, based of a few guidelines available at that time.  **Verbal learning:** assessed using the Hopkins Verbal Learning Test with Crawford Alternative [11], using the number if items recalled on trial.  **Vigilance:** assessed with the Continuous Performance Test [12]  **Speed**: assessed using a Groove Pegboard test through the number of pegs successfully inserted on trial.  **Reasoning:** measures using the number of completed categories in the *Wisconsin Card Sorting Test* [13].  **Working memory:** measured using the *Computerized test of visuospatial working memory* [14].  **Total score:** Neurocognitive Composite Score Created Variable Standardized to baseline. |
| Antipsychotic medication & Adverse Events | |
| Exposition to study medication^b^   - Olanzapine - Quetiapine - Risperidone - Perphenazine - Clozapine - Fluphenazine - Aripiprazole | Dichotomic, took that study medication at some point between the baseline and the 6 months visit (1) or not (0). |
| Number of days exposed to study medication^b^   - Olanzapine - Quetiapine - Risperidone | Continuous scale representing the number of days between baseline and visit 6 during which the patient took that study medication. A higher value means that the patient has been more exposed (assuming they were adherent). |

**Supplementary Table. Variables included into database and computed using supervised machine learning.** *(continued)*

| Variables | Description |
| --- | --- |
| Antipsychotic medication & Adverse Events *(continued)* | |
| Number of days exposed to study medication^b^ *(continued)*   - Perphenazine - Clozapine - Fluphenazine - Aripiprazole |  |
| Adherence to study medication ^b^   1. Has the patient taken the study medication? 2. Clinical judgment of medication adherence | Item (a) : dichotomic, patient is adherent (1) vs. non-adherent (0).  This item was based on the question answered by the physician : “has patient taken medication using dosage frequency by [the randomization system]?”  Item (b) : ordinal, a higher value meaning the patient is less adherent   1. Always / almost always [76-100% of the time] 2. Usually [51-75% of the time] 3. Sometimes [26-50% of the time] 4. Never / almost never [0-25% of the time] |
| Severity of adverse event according to the physician:   - Orthostatic faintness - Dry mouth - Constipation - Sialorrhea - Gynecomastia - Sex drive - Sexual arousal - Sexual orgasm - Incontinence/Nocturia - Urinary hesitancy - Skin rash - Sleepiness - Hypersomnia - Insomnia - Weight gain - Akathisia - Akinesia | The adverse events were systematically inquired during each visit through an Adverse Events Side Effects questionnaire.  Ordinal:   1. Absent 2. Mild 3. Moderate 4. Severe   *Note: the adverse event “menstrual irregularities” has been removed from the dataset because it was not applicable to most of the sample. |

**Supplementary Table. Variables included into database and computed using supervised machine learning.** *(continued)*

| Variables | Description |
| --- | --- |
| Antipsychotic medication & Adverse Events *(continued)* | |
| Severity of adverse event according to the patient:   - Orthostatic faintness - Dry mouth - Constipation - Sialorrhea - Menstrual irregularities - Gynecomastia - Sex drive - Sexual arousal - Sexual orgasm - Incontinence/Nocturia - Urinary hesitancy - Skin rash - Sleepiness - Hypersomnia - Insomnia - Weight gain - Akathisia - Akinesia | The adverse events were systematically inquired during each visit through an Adverse Events Side Effects questionnaire.  Ordinal:   1. Absent 2. Mild 3. Moderate 4. Severe |
| Impact of adverse event on patients’ adherence to study medication   - Orthostatic faintness - Dry mouth - Constipation - Sialorrhea - Menstrual irregularities - Gynecomastia - Sex drive - Sexual arousal - Sexual orgasm - Incontinence/Nocturia - Urinary hesitancy - Skin rash - Sleepiness - Hypersomnia - Insomnia - Weight gain - Akathisia - Akinesia | The adverse events were systematically inquired during each visit through an Adverse Events Side Effects questionnaire.  Ordinal:   1. Unrelated to antipsychotic medication 2. Patient thinks SE caused by med - no impact on adherence to med 3. Patient thinks SE caused by med and SE affects willingness to take med- but is still taking med 4. Pt thinks SE caused by med and is not willing to take medication due to SE |

**Supplementary Table. Variables included into database and computed using supervised machine learning.** *(continued)*

| Variables | Description |
| --- | --- |
| Physical health | |
| Vitals   - Body mass index - Body mass index categorized - Height - Systolic blood pressure - Diastolic blood pressure - Waist - Hips - Waist/hips ratio - Heart rate - Weight | Vital signs were measured regularly.  Continuous variables using standard units (imperial system) |
| Lab values   - Albumin (g/dL) - Alkaline phosphatase (IU/L) - ALT SGPT (IU/L) - AST SGOT (IU/L) - Bicarbonate (meq/L) - Bun (mg/dL) - Calcium (mg/dL) - Chloride (meq/L) - CPK (IU/L) - Creatinine (mg/dL) - GGT (mg/dL) - Glucose (mg/dL) - Phosphorus (mg/dL) - Potassium (meq/L) - Sodium (meq/L) - Total bilirubin (mg/dL) - Total protein (g/dL) - Uric acid (mg/dL) - Basophils (%) - Eosinophils (%) - Hematocrit (%) - Hemoglobin (g/dL) - Lymphocytes (%) - MCH (PG/cell) - MCHC (GHB/cell) - MCV (FL) - Monocytes (%) - Neutrophils (%) - Platelet count (x10E3/uL) | Laboratory tests were conducted, including clinical chemistry and electrolytes (sodium, potassium, bicarbonate, chloride, blood urea nitrogen, creatinine, phosphorus, uric acid, calcium, total protein, albumin, creatinine kinase, alkaline phosphatase), liver function tests (GGT, AST, ALT, total bilirubin), thyroid stimulating hormone, complete blood count, urinalysis, fasting blood glucose, lipids and (total cholesterol, HDL cholesterol and triglycerides). Only values that were measured for most patients were included in the present database. |

**Supplementary Table. Variables included into database and computed using supervised machine learning.** *(continued)*

| Variables | Description |
| --- | --- |
| Physical health *(continued)* | |
| Lab values *(continued)*   - RBC (x10E6/uL) - HDL cholesterol (mg/dL) - Total cholesterol (mg/dL) - Triglycerides (mg/dL) |  |

^a^ Only measured at baseline, and is therefore not included in model 2.

^b^ Not measured at baseline or N/A for baseline, and therefore is not included in models 1 and 3.

1. Glasofer, D.R., A.J. Brown, and M. Riegel, *Structured Clinical Interview for DSM-IV (SCID)*, in *Encyclopedia of Feeding and Eating Disorders*, T. Wade, Editor. 2015, Springer Singapore: Singapore. p. 1-4.

2. Busner, J. and S.D. Targum, *The clinical global impressions scale: applying a research tool in clinical practice.* Psychiatry (Edgmont (Pa. : Township)), 2007. **4**(7): p. 28-37.

3. Addington, D., J. Addington, and B. Schissel, *A depression rating scale for schizophrenics.* Schizophr Res, 1990. **3**(4): p. 247-51.

4. Addington, J., et al., *Reliability and validity of the Calgary Depression Scale for Schizophrenia (CDSS) in youth at clinical high risk for psychosis.* Schizophrenia Research, 2014. **153**(1): p. 64-67.

5. Amri, I., A. Millier, and M. Toumi, *Minimum Clinically Important Difference in the Calgary Depression Scale for Schizophrenia.* Value Health, 2014. **17**(7): p. A766.

6. Awad, A.G., *Subjective response to neuroleptics in schizophrenia.* Schizophr Bull, 1993. **19**(3): p. 609-18.

7. McEvoy, J.P., et al., *Insight in schizophrenia. Its relationship to acute psychopathology.* J Nerv Ment Dis, 1989. **177**(1): p. 43-7.

8. Steadman, H.J., et al., *Violence by people discharged from acute psychiatric inpatient facilities and by others in the same neighborhoods.* Arch Gen Psychiatry, 1998. **55**(5): p. 393-401.

9. Kay, S.R., A. Fiszbein, and L.A. Opler, *The positive and negative syndrome scale (PANSS) for schizophrenia.* Schizophr Bull, 1987. **13**(2): p. 261-76.

10. Ware, J., Jr., M. Kosinski, and S.D. Keller, *A 12-Item Short-Form Health Survey: construction of scales and preliminary tests of reliability and validity.* Med Care, 1996. **34**(3): p. 220-33.

11. Benedict, R.H.B., et al., *Revision of the Brief Visuospatial Memory Test: Studies of normal performance, reliability, and validity.* Psychological Assessment, 1996. **8**(2): p. 145-153.

12. Cornblatt, B.A., et al., *The continuous performance test, identical pairs version (CPT-IP): I. new findings about sustained attention in normal families.* Psychiatry Research, 1988. **26**(2): p. 223-238.

13. Heaton, R., *Wisconsin card sorting test manual; revised and expanded.* Psychological Assessment Resources, 1981: p. 5-57.

14. Hershey, T., et al., *Spatial long-term but not working memory decreases over time in schizophrenia.* Society for Neuroscience Abstracts, 1999. **25**(Part 1): p. 572.
