# Supplementary material for: Prediction of quality of life in schizophrenia using machine learning models on data from Clinical Antipsychotic Trials of Intervention Effectiveness (CATIE) schizophrenia trial
Source: Schizophrenia (Heidelb). 2022 Mar 21;8(1):29. doi: 10.1038/s41537-022-00236-w (PMC8938459; doi:10.1038/s41537-022-00236-w)
Supplement: Supplementary file 3 — Supplementary Method. Pseudocode [file 41537_2022_236_MOESM3_ESM.docx]

**Supplementary Method. Pseudocode**

## 1. Data splitting

X = values of the predictors of the dataset

y = dependent variables of the dataset

X training set, X testing set, y training set, y testing set = train test split function(X, y, size of the test = 30%, selection of the random state)

## 2. LASSO and GridSearchCV nesting.

LASSO_model = Lasso(Randome State, Maximum iteration)

alpha values = log space of various alphas

tuned_parameters = [{'alpha': alphas}]

n_folds = 10

search=GridSearchCV (model_lasso, tuned_parameters, CV = n_folds)

search.fit(X training set, y training set)

LASSO_model.set_params(**search.best_params_)

LASSO_model.fit(X training set, y training set)

pred_train_lasso= model_lasso.predict(X training set)

numpy.sqrt(mean_squared_error(y training set,pred_train_lasso))

r2_score_train(y training set, pred_train_lasso)

pred_test_lasso= model_lasso.predict(X testing set)

numpy.sqrt(mean_squared_error(y testing set,pred_test_lasso)

r2_score_test(y testing set, pred_test_lasso)
